# Fabrication and Characterization of Photovoltaic Microgenerators Using the Complementary Metal Oxide Semiconductor Process

**DOI:** 10.3390/mi14112038

**Published:** 2023-10-31

**Authors:** Chun-Yu Chen, Zhi-Xuan Dai

**Affiliations:** 1 Department of Mechanical Engineering, National Chung Hsing University, Taichung 402, Taiwan; 2 Department of Bio-Industrial Mechatronics Engineering, National Chung Hsing University, Taichung 402, Taiwan

**Keywords:** photovoltaic microgenerator, energy-conversion efficiency, fill factor, MEMS, CMOS

## Abstract

This study develops a photovoltaic microgenerator based on the complementary metal oxide semiconductor (CMOS) process. The photovoltaic microgenerator converts the absorbed light energy into electrical energy using the photovoltaic effect. The material for the photovoltaic microgenerator is silicon, and its structure consists of patterned p–n junctions. The design of the photovoltaic microgenerator utilizes a grid-like shape, forming a large-area p–n junction with a patterned p-doping and N-well structure to enhance the photocurrent and improve the device’s performance. The photovoltaic microgenerator is fabricated employing the CMOS process with post-processing step. Post-processing is applied to enhance the microgenerator’s light absorption and energy-conversion efficiency. This involves using wet etching with buffered-oxide etch (BOE) to remove the silicon dioxide layer above the p–n junctions, allowing direct illumination of the p–n junctions. The area of the photovoltaic microgenerator is 0.79 mm^2^. The experimental results show that under an illumination intensity of 1000 W/m^2^, the photovoltaic microgenerator exhibits an open-circuit voltage of 0.53 V, a short-circuit current of 233 µA, a maximum output power of 99 µW, a fill factor of 0.8, and an energy-conversion efficiency of 12.5%.

## 1. Introduction

With the rapid advancement of technology, the human demand for energy has grown exponentially. Currently, the primary energy source is derived from the combustion of fossil fuels, leading to substantial emissions of greenhouse gases and severe environmental consequences. To alleviate this burden, the promotion of alternative green energy sources has become paramount. Green energy taps into the Earth’s inexhaustible natural resources and is renewable, encompassing solar, hydro, wind, and geothermal power. Among these renewable sources, solar energy stands out for its unique capacity to transform sunlight into electricity, relying solely on light exposure for power generation [1,2,3,4,5,6]. Despite the array of available green energy sources, solar energy remains a prominent contender. However, most commercially available solar generators are characterized by their large size, rendering them unwieldy and lacking in mobility. Photovoltaic microgenerators improve these limitations and have many applications. For instance, in the work of Qiu et al. [7], nanocarbon tubes and solution processes were utilized to fabricate flexible, fiber-like perovskite microsolar generators. These were seamlessly integrated with textile technology to produce electronic textile products capable of powering garments autonomously. Similarly, Hyun et al. [8] integrated photovoltaic microgenerators with portable light-emitting diode (LED) lights, enabling daytime energy absorption and nighttime illumination. This microphotovoltaic system converts light energy into electricity, powering portable LED lights for both indoor and outdoor use.

Photovoltaic generators were created using microelectromechanical system (MEMS) technology, offering benefits such as size reduction, seamless integration with IC devices, and suitability for large-scale manufacturing. Several photovoltaic microgenerators have been developed using MEMS and CMOS technologies. For instance, Sakai et al. [9] fabricated organic solar cells utilizing heterojunction organic materials through screen printing. The materials used in the organic solar cell included the conjugated polymers as donors and the derivatives of fullerenes as acceptors. The results indicated that under AM1.5G solar irradiation, an open-circuit voltage of 0.821 V and an energy-conversion efficiency of 2.35% were achieved. However, this cost-effective screen-printing method often leads to uneven material distribution, resulting in variable performance among different solar cells and, ultimately, lower-than-anticipated efficiency. Arima et al. [10] employed the CMOS process to develop solar cells. They established two distinct p–n junctions by doping p-type and n-type silicone onto the solar chip, subsequently serially connecting these junctions. The results showed that an irradiance of 2000 lx led to an output voltage of 0.83 V and an energy-conversion efficiency of 2.6%. Marwick et al. [11] employed sapphire CMOS technology to manufacture photovoltaic generators. Sapphire, serving as an insulating substrate, was utilized to enhance the transmission of light from the rear. By serially connecting multiple sets of photodiodes, a high output voltage was attained. The materials used in the photodiodes were doped p-type and n-type silicon. The device revealed that an irradiance of 5000 lx resulted in a power output of 60 mW. Across the range of wavelengths from infrared to ultraviolet, the photovoltaic generator demonstrated an energy-conversion efficiency that was greater than 1%. Despite transitioning to transparent sapphire material from the traditional substrate, the energy-conversion efficiency for the device was not high. Law et al. [12] manufactured cascaded photodiodes using the CMOS process. The materials employed in the photodiodes were doped p-type and n-type silicon. The study employed second- and third-level cascading, yielding open-circuit voltages of 0.84 V and 1.3 V accompanied by short-circuit currents of 1.25 nA and 1.12 nA and conversion efficiencies of 0.3% and 0.06%, respectively, under an irradiance of 6.37 mW/cm^2^. Although the study solely compared second and third-level cascading of photodiodes and suggested the superiority of third-level over second-level cascading, it failed to discuss the design of p–n junctions. Consequently, improvement in energy-conversion efficiency is still necessary. Horiguchi et al. [13] produced lightly doped triple-layer solar cells using CMOS technology. The interconnected triple-layer light doping augmented the output voltage and mitigated leakage current issues. Experiments demonstrated that under an irradiance of 31,000 lx, a substantial open-circuit voltage of 1.3 V, coupled with a short-circuit current of 6 µA and an energy-conversion efficiency of 7.5%, was achieved. Despite achieving a remarkable open-circuit voltage through series connection, the current enhancement was inadequate and, thus, still had a low energy-conversion efficiency. Hung et al. [14] fabricated interdigitated back-contact (IBC) solar cells using the CMOS process. By significantly reducing the thickness of the silicon substrate, the energy-conversion efficiency was elevated from 4% to 15%. Under conditions involving a wavelength of 980 nm and an irradiance of 10 mW/m^2^, an open-circuit voltage of 0.57 V and an output power of 159 μW were obtained. While thinning the silicon substrate indeed improved performance substantially, the technical complexity and cost of this approach have limited its widespread adoption. Hung et al. [15] manufactured photovoltaic chips by selectively removing portions of the substrate using CMOS processes. That study managed to enhance the output voltage of back-illuminated photovoltaic chips by employing a substrate removal approach and stacking photovoltaic structures. Under an intensity of 40 mW, an open-circuit voltage of 2.05 V, a short-circuit current of 2.6 mA, and an energy-conversion efficiency of 11% were achieved. Hung et al. [16] developed photovoltaic microgenerators, segregating photovoltaic units on the chip to form photovoltaic modules. The materials used in the photovoltaic microgenerators were doped p-type and n-type silicon. Experimental results revealed an open-circuit voltage of 0.5 V for a single photovoltaic unit. The series connection of 25 units resulted in an open-circuit voltage of 12.5 V, a short-circuit current of 11 µA, and an energy-conversion efficiency of 0.5% under an irradiance of 6 mW/mm^2^. Despite attaining a substantial 12.5 V output voltage, the 0.5% energy-conversion efficiency was insufficient to enable effective self-power generation. Therefore, increasing the output current of the microgenerator became imperative. Yan et al. [17] used MEMS technology to fabricate thermoelectric–photovoltaic generators, combining thermoelectric microgenerators with photovoltaic counterparts. Photovoltaic elements adopted interdigitated electrode structures, incorporated beneath the thermoelectric structure. The materials of the photovoltaic microgenerators were doped p-type and n-type silicon. The experimental results demonstrated a fill factor of 0.792 and an energy-conversion efficiency of 4.45% when the photovoltaic component was illuminated with an irradiance of 100 mW/cm^2^. While the interdigitated electrode structure contributed to enhance photovoltaic performance, the stack configuration of the thermoelectric–photovoltaic generator led to performance interference. Sichao et al. [18] developed thermoelectric–photovoltaic microgenerators incorporating metal heat sinks through the MEMS process. This integration combined thermoelectric microgenerators with photovoltaic counterparts. The photovoltaic microgenerator employed a back-contact structure, allowing for autonomous selection of the illuminated surface. The materials utilized in the photovoltaic microgenerators were doped p-type and n-type silicon. Under illumination with an irradiance of 100 mW/cm^2^, the photovoltaic microgenerator achieved an energy-conversion efficiency of 5.446%. While the choice of illuminated surface was feasible, challenges associated with structural integration gave rise to mutual interference, subsequently undermining the photovoltaic performance and energy-conversion efficiency. Collectively, the aforementioned literature underscores the recent trend in photovoltaic microgenerators. These studies have explored altered silicon substrate structures, embraced back-illuminated configurations, and endeavored to fuse photovoltaic structures with alternative power-generation arrangements to achieve heightened output voltages. Nevertheless, it is evident that the performance of photovoltaic microgenerators can also be enhanced by means of patterned designs and alterations in the process materials through doping. This study focuses on the patterned design of p–n junctions within a photovoltaic microgenerator to enhance its performance.

Various microsensors [19,20,21,22,23], actuators [24,25,26,27,28,29], and devices [30,31,32,33,34] have been fabricated using the CMOS-MEMS technique. Photovoltaic microgenerators created through this technique offer improved efficiency, compactness, versatility, and cost-effectiveness, making them well-suited for a wide range of applications [35,36,37,38,39], including self-powered sensors, wearable devices, and internet of things (IoT). In this study, the same technique is employed to design a photovoltaic microgenerator. Additionally, a post-processing step is utilized to etch the silicon dioxide layer of the photovoltaic microgenerator, thereby enhancing its performance. In this paper, the design of the structure, simulation of its characteristics, the fabrication process, and the testing results of the photovoltaic microgenerator will be detailed.

## 2. Design of the Photovoltaic Microgenerator

A photovoltaic generator is a semiconductor device that converts sunlight directly into electrical energy through the photovoltaic effect. The fundamental principle behind the operation of a photovoltaic generator involves the interaction of light with semiconductor materials to generate an electric current. In this study, the material of the photovoltaic generator is silicon. When sunlight, which is composed of photons, strikes the surface of the silicon, it transfers energy to the atoms within the material. This energy is sufficient to dislodge electrons from their positions in the atoms, creating electron–hole pairs. The silicon material is doped with impurities to create a p–n junction. This is a boundary between a region with excess electrons (n-type) and a region with excess holes (p-type). The difference in charge concentration creates an electric field across the junction. The p–n junction absorbs photons, generating electrons and holes. Electrons are directed toward the n-type region, while holes are directed toward the p-type region. This charge separation leads to the accumulation of a voltage difference across the p–n junction. When an external electrical circuit is connected to the photovoltaic generator, the separated charges flow through the circuit to recombine on the other side of the p–n junction. This movement of charges constitutes an electric current, which can be used to power electronic devices or stored in batteries.

**Figure 1:**
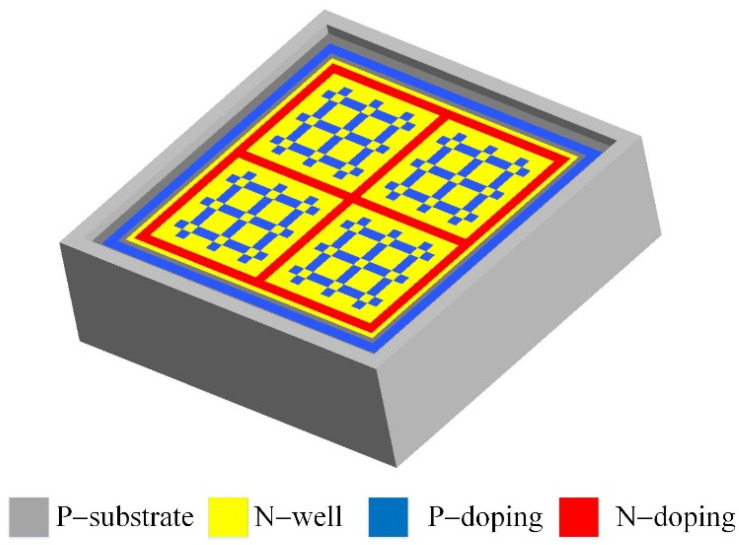
Three-dimensional structure of the photovoltaic microgenerator.

Figure 1 illustrates the three-dimensional structure of the photovoltaic microgenerator. By incorporating distinct p-type and n-type doping into a silicon substrate, the photovoltaic microgenerator gives rise to intricate semiconductor configurations that involve p-type doping, n-type light doping (N-well), and n-type doping. These intricate configurations are structured to yield the photovoltaic microgenerator’s functional framework. In this research, the patterned design is characterized by a mesh format, setting it apart from the conventional interdigitated shape layouts. As depicted in Figure 1, an extensive n-type lightly doped structure is seamlessly integrated into the silicon substrate. This strategic employment of light doping serves a dual purpose: it mitigates leakage currents while concurrently amplifying the performance of the p–n junctions. Moreover, a mesh-patterned p-type doping is ingeniously embedded within the region featuring n-type light doping. This innovative amalgamation of attributes—encompassing p-type doping/n-type light doping as well as p-type silicon substrate/n-type light doping p–n junctions—effectively enhances the power-generation capabilities of the photovoltaic microgenerator.

This research utilized the Sentaurus TCAD 2021 semiconductor device-simulation software developed by Synopsys to analyze the photovoltaic microgenerator. As shown in Figure 1, the model of the photovoltaic microgenerator is established, and the TCAD simulation process comprises three main stages: modeling and configuring the material parameters, meshing the model, and performing the computational analysis. In the initial phase, a three-dimensional model is constructed employing the Sentaurus structure-editor (SDE) tool, a component of the TCAD software. SDE delineates the structure of the model with fixed coordinates, delineating its dimensions, doping parameters across diverse layers, positioning the electrodes, and defining the Gaussian distribution to replicate the diffusion phenomena encountered during the actual chip fabrication. The subsequent phase encompasses mesh generation, entailing the determination of the model’s mesh dimensions. Following the completion of meshing, the simulation’s boundary conditions and environmental variables are established through command and parameter files. The command file governs environmental variables encompassing temperature, irradiance, light positioning, and wavelength. On the other hand, the parameter file manages the specification of the materials and physical property parameters. Once these variables are configured, the third step involves computing and analyzing the outcomes.

**Figure 2:**
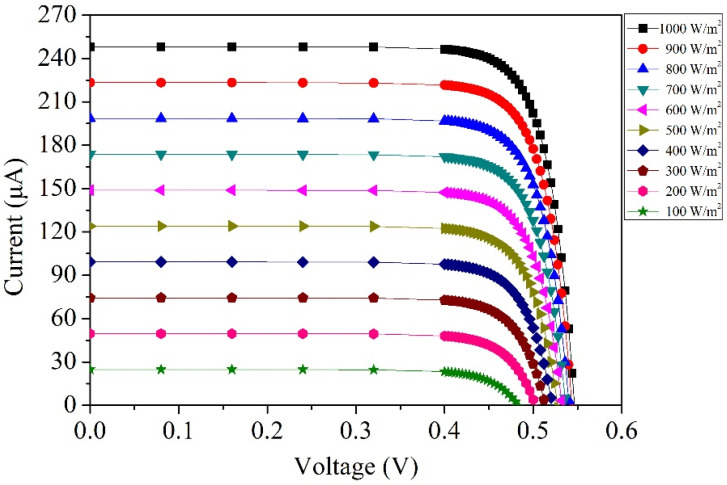
Simulated voltage–current characteristics of the photovoltaic microgenerator.

Figure 2 depicts the simulated voltage–current characteristics of the photovoltaic microgenerator under varying irradiance levels. Maintaining a constant wavelength and temperature, the simulation explores the voltage–current relationship across irradiance levels ranging from 100 W/m^2^ to 1000 W/m^2^, at 100 W/m^2^ intervals. The simulations were carried out at a temperature of 25 °C and a wavelength of 600 nm. As illustrated in Figure 2, at an irradiance of 100 W/m^2^, the open-circuit voltage is 0.48 V, while the short-circuit current registers at 27.5 µA. As the irradiance escalates to 1000 W/m^2^, the open-circuit voltage elevates to 0.55 V, accompanied by a rise in the short-circuit current to 248 µA. The simulated results show that higher irradiance levels increase the current values of the photovoltaic generator.

**Figure 3:**
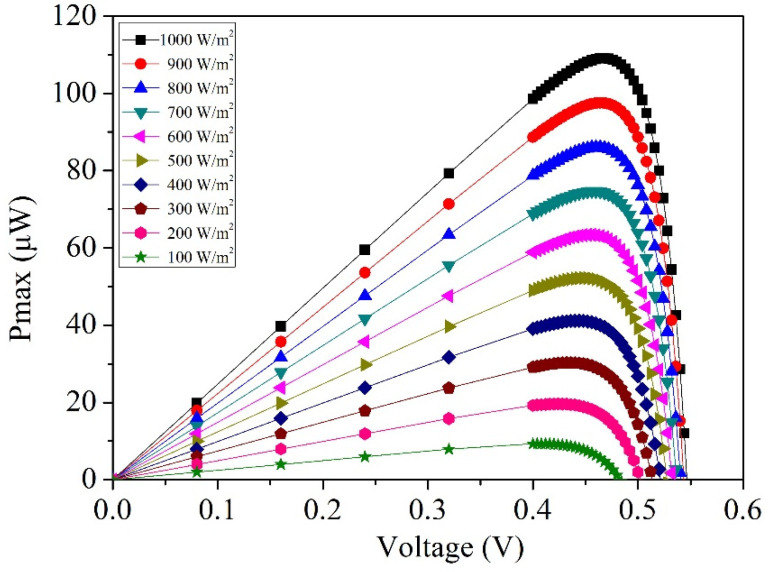
Simulated output power of the photovoltaic microgenerator.

Output power stands as a critical performance metric for photovoltaic generators. The output power (*P_o_*) of a photovoltaic microgenerator can be expressed as follows [40]:

(1) $$P_{o}=I_{o}V_{o}$$

where *I_o_* represents the output current of the photovoltaic microgenerator, and *V_o_* is its output voltage. When the output current and voltage of the photovoltaic microgenerator are known, Equation (1) becomes instrumental in computing its output power. The simulation results of current and voltage for the photovoltaic microgenerator are depicted in Figure 2. By substituting these simulated outcomes into Equation (1), it becomes feasible to calculate the output power of the photovoltaic microgenerator, as depicted in Figure 3. The simulated output power of the photovoltaic microgenerator is shown in Figure 3. At an irradiance of 100 W/m^2^, with an output voltage of 0.43 V, the photovoltaic microgenerator achieves a peak output power of 8.5 µW. As the irradiance increases to 1000 W/m^2^, with an output voltage of 0.47 V, the photovoltaic microgenerator reaches its maximum output power of 109 µW.

The fill factor (*FF*) of a photovoltaic microgenerator is a measure of its ability to convert sunlight into electricity efficiently. It quantifies the quality of the current–voltage (I-V) characteristics of the photovoltaic microgenerator. The *FF* of the photovoltaic microgenerator is defined as [14]:

(2) $$FF=\frac{P_{max}}{V_{oc}I_{sc}}$$

where *P_max_* is the maximum output power of the photovoltaic microgenerator, *V_oc_* is the open-circuit voltage of the photovoltaic microgenerator, and *I_sc_* is the short-circuit current of the photovoltaic microgenerator. A higher fill factor indicates better performance and efficiency in converting sunlight into usable electrical power. As shown in Figure 2 and Figure 3, at an irradiance of 1000 W/m^2^, the photovoltaic microgenerator has an open-circuit voltage of 0.55 V, a short-circuit current of 248 µA, and a maximum output power of 109 µW. By substituting these values into Equation (2), the evaluated *FF* of the photovoltaic microgenerator is 0.8.

The energy-conversion efficiency of a photovoltaic microgenerator refers to the ratio of the electrical power output generated by the photovoltaic device to the incident solar energy (light) input from the sun. It represents the effectiveness of the photovoltaic microgenerator in converting sunlight into usable electrical energy. The energy-conversion efficiency (*η*) of the photovoltaic microgenerator is expressed as [14]:

(3) $$\eta =\frac{P_{max}}{P_{in}}\times 100\%=\frac{P_{max}}{AE}\times 100\%$$

where *P_max_* is the maximum output power of the photovoltaic microgenerator, *P_in_* is the input power of the incident light, *A* is the area of the photovoltaic microgenerator, and *E* is the irradiance of the incident light. As shown in Figure 3, at an irradiance of 1000 W/m^2^, the maximum output power of the photovoltaic microgenerator is 109 µW. The area of the photovoltaic microgenerator is 0.79 mm^2^. By substituting these values into Equation (3), the evaluated energy-conversion efficiency of the photovoltaic microgenerator is 13.8%.

## 3. Fabrication of the Photovoltaic Microgenerator

**Figure 4:**
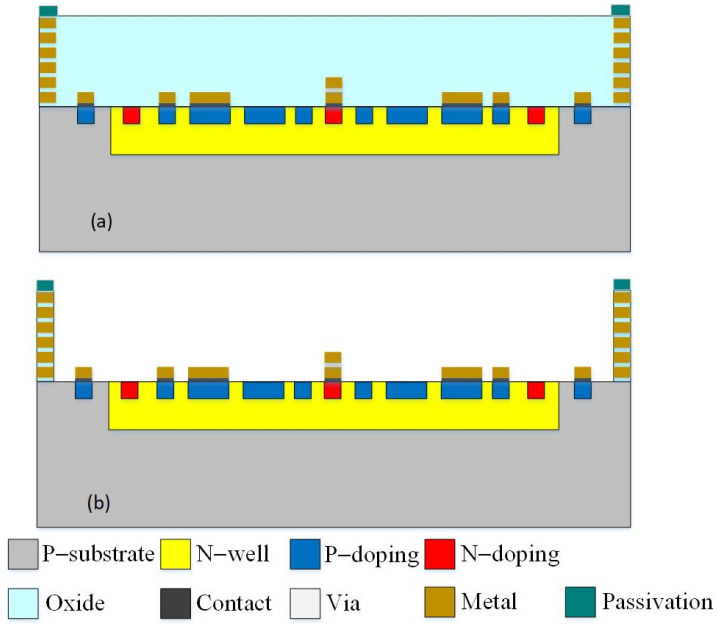
Cross-sectional structure of the photovoltaic microgenerator: (**a**) completion of the completion of the CMOS process; (**b**) etching the silicon dioxide layer.

The photovoltaic microgenerator is produced utilizing the standardized 0.18 μm CMOS process of Taiwan Semiconductor Manufacturing Company (TSMC). TSMC executed the fabrication of the photovoltaic microgenerator in accordance with the layout design depicted in Figure 1, utilizing the CMOS process. Figure 4 illustrates the process flow of the photovoltaic microgenerator. The oxide layer is initially present as part of the standard CMOS process. Its primary purpose is to act as a dielectric layer or a passivation layer. The passivation layer protects the underlying structures from environmental factors and potential contaminants. Figure 4a presents the cross-sectional structure of the photovoltaic microgenerator after the completion of the CMOS process. Notably, a silicon dioxide layer overlays the p–n junctions. To mitigate potential photon energy losses due to the reflections, refractions, or absorptions caused by the silicon dioxide layer—factors that could potentially impede the power generation efficiency of the photovoltaic microgenerator—this study employs a post-processing technique to remove the silicon dioxide layer.

**Figure 5:**
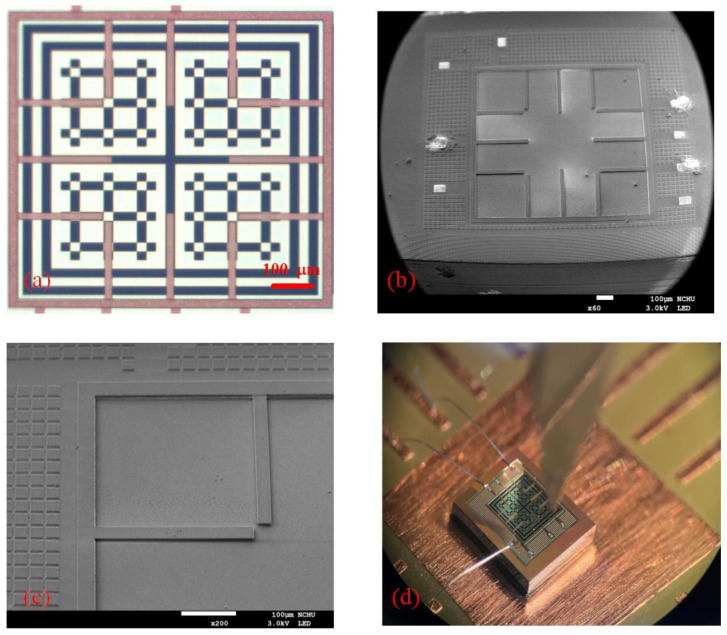
The photovoltaic microgenerator: (**a**) optical image of following the completion of the CMOS process; (**b**) scanning electron microscope (SEM) image after etching the silicon dioxide layer; (**c**) localized SEM image after post-processing; (**d**) image of the photovoltaic microgenerator chip during the wire bonding.

Figure 4b shows the cross-sectional constitution of the photovoltaic microgenerator following the removal of the silicon dioxide layer. Post-processing involves the utilization of a BOE solution to etch the silicon dioxide layer until the p–n junctions are exposed. The thickness of the oxide layer was monitored using ellipsometry measurements to ensure the complete removal of the oxide layer during the etching process. The measured thickness of the oxide layer was about 8 μm. This configuration, as illustrated in Figure 4b, enables direct illumination of the p–n junctions by incident light, enhancing the overall efficiency. Figure 5a illustrates the optical image of the photovoltaic microgenerator following the completion of the CMOS process. In Figure 5b, a scanning electron microscope (SEM) image of the photovoltaic microgenerator after silicon dioxide layer etching is depicted. The SEM image distinctly reveals substantial depressions on the surface of the photovoltaic microgenerator’s structure resulting from the etching process. Figure 5c provides a localized SEM image that accentuates the concave characteristics resulting from the etching process, thus confirming the elimination of the silicon dioxide layer. The etching process is carefully optimized to minimize any adverse effects on the photovoltaic microgenerator chip. The SEM images (Figure 5) and the testing of the photovoltaic microgenerator demonstrate that the etching process does not compromise the structural integrity or performance of the photovoltaic microgenerator. The removal of the oxide layer can potentially expose the underlying material to external influences, which might reduce the device’s reliability. Figure 5d displays the image of the photovoltaic microgenerator chip subsequent to the wire-bonding procedure. The chip was affixed to a printed circuit board (PCB), and a wire bonder was employed to establish connections between the pads on the photovoltaic microgenerator chip and the PCB, utilizing aluminum wires.

## 4. Results

The performance assessment of the photovoltaic microgenerator involved the utilization of a testing chamber, an optical power meter, and a digital multimeter. Within the testing chamber, a tungsten filament lamp served as the incident light source, offering adjustable irradiance through integrated control knobs. The tungsten filament lamp (TFC 250 W 120 V, Taiwan Fluorescent Lamp Co., Taipei, Taiwan) has a spectral response of approximately 380–780 nm. The optical power meter was utilized for calibrating the illumination intensity within the chamber. The digital multimeter was deployed to measure the output voltage and current generated by the photovoltaic microgenerator. The photovoltaic microgenerator was set within the testing chamber, and the intensity of the incident light source was regulated by manipulating the irradiance control knobs. The optical power meter was employed to monitor the irradiance level of the incident light source, whereas the digital multimeter recorded the output voltage and current of the photovoltaic microgenerator.

**Figure 6:**
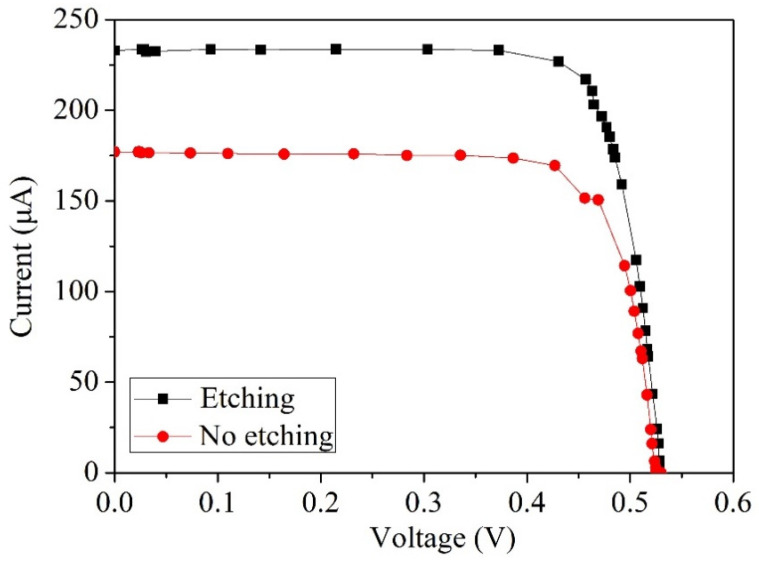
Current–voltage characteristics of photovoltaic microgenerators with etched and unetched silicon dioxide layers.

To comprehend the performance differences between photovoltaic microgenerators featuring etched and unetched silicon dioxide layers, tests were performed on both the photovoltaic microgenerator with an unetched silicon dioxide layer and the one with an etched silicon dioxide layer. Figure 6 illustrates the current–voltage curves of the photovoltaic microgenerators with etched and unetched silicon dioxide layers. These measurements were taken at room temperature with an incident light-source irradiance of 1000 W/m^2^.

As shown in Figure 6, the short-circuit current of the photovoltaic microgenerator with an unetched silicon dioxide layer is 175 µA. In comparison, the photovoltaic microgenerator with an etched silicon dioxide layer exhibits a 25% increase in short-circuit current. This increase can be attributed to the fact that the incident light can directly illuminate the p–n junctions without being obstructed or interfered with by the silicon dioxide layer in the case of the photovoltaic microgenerator with an etched silicon dioxide layer. Consequently, the short-circuit current of the photovoltaic microgenerator with an etched silicon dioxide layer is enhanced. The influence of the silicon dioxide layer on light absorption in the photovoltaic microgenerator is primarily related to the optical properties and surface interactions. While SiO_2_ itself is relatively transparent in the visible and near-infrared regions due to its high bandgap, its presence can still impact light absorption in a few ways. Firstly, the silicon dioxide layer can cause reflection and refraction of the incident light, leading to losses in the overall light absorption. Additionally, any surface imperfections or roughness induced by the silicon dioxide layer might contribute to scattering or diffraction of the light, thereby reducing the efficiency of light capture by the photovoltaic material. It is important to note that while SiO_2_ may not directly absorb light, its presence can indirectly influence the overall light absorption efficiency of the photovoltaic microgenerator through these optical phenomena.

**Figure 7:**
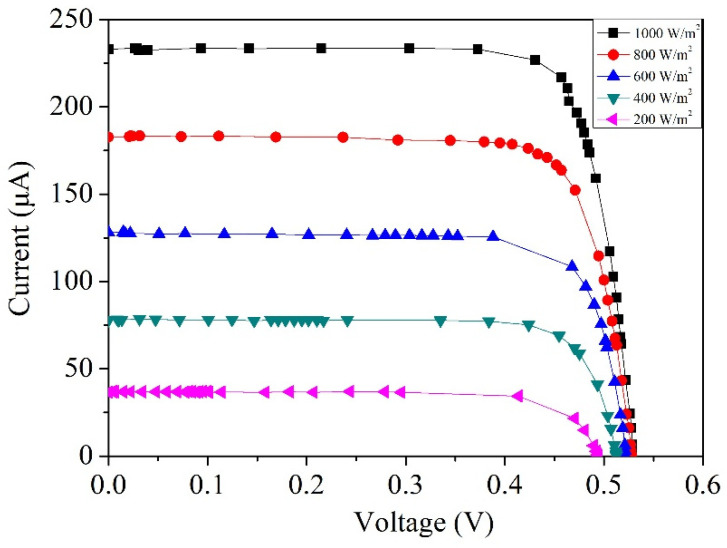
Measured voltage–current characteristics of the photovoltaic microgenerator.

The measurement results from Figure 6 indicate that the performance of the photovoltaic microgenerator with the etched silicon dioxide layer surpasses that of the one with an unetched silicon dioxide layer. Consequently, all subsequent tests were conducted using the photovoltaic microgenerator with the etched silicon dioxide layer. Figure 7 illustrates the voltage–current curves of the photovoltaic microgenerator measured at various irradiance levels. These measurements were carried out at intervals of 200 W/m^2^, encompassing irradiance levels ranging from 200 W/m^2^ to 1000 W/m^2^. As depicted in Figure 7, at an irradiance of 200 W/m^2^, the open-circuit voltage is 0.49 V, accompanied by a short-circuit current of 38 µA. With an increase in irradiance to 1000 W/m^2^, the open-circuit voltage ascends to 0.53 V, while the short-circuit current reaches 233 µA.

**Figure 8:**
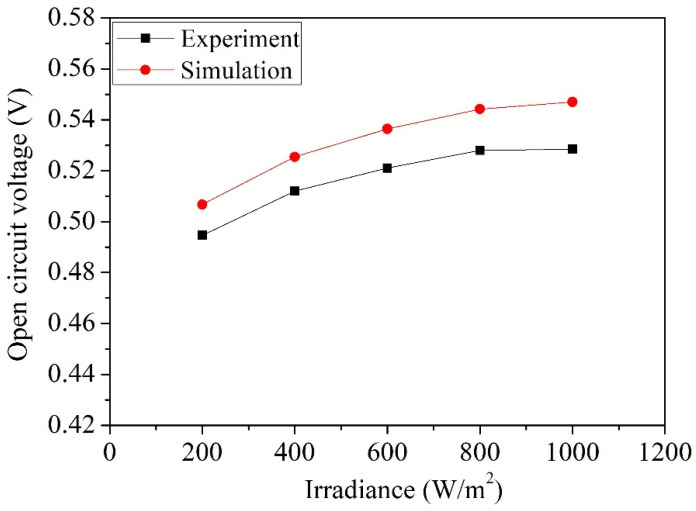
Open-circuit voltage of the photovoltaic microgenerator as a function of the irradiance.

**Figure 9:**
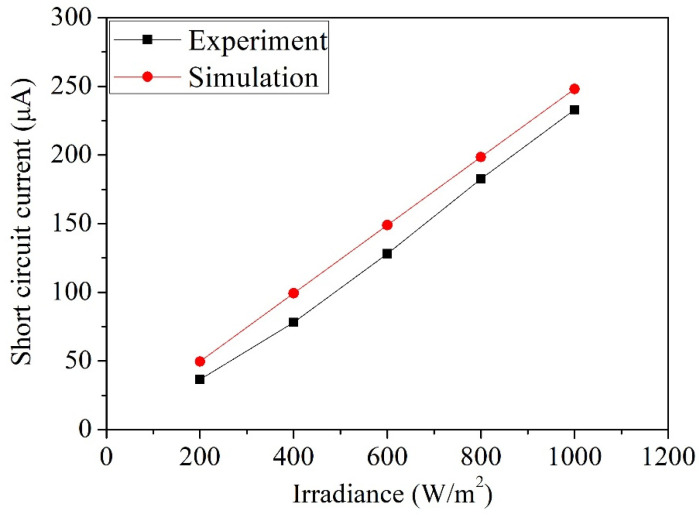
Short-circuit current of the photovoltaic microgenerator as a function of the irradiance.

Figure 8 illustrates the comparison between the simulated and measured open-circuit voltages of the photovoltaic microgenerator. The simulated results are based on the open-circuit voltage data obtained from the photovoltaic microgenerator under different irradiance levels, as shown in Figure 2. On the other hand, the measured results are gathered from the open-circuit voltage data recorded under varying irradiance levels, as depicted in Figure 7. As depicted in Figure 8, at an irradiance of 200 W/m^2^, the simulated open-circuit voltage is 0.51 V, while the measured open-circuit voltage is 0.49 V. When the irradiance increases to 1000 W/m^2^, the simulated open-circuit voltage is 0.55 V, and the measured open-circuit voltage is 0.53 V. The comparison of simulated and measured open-circuit voltages of the photovoltaic microgenerator reveals an error percentage of approximately 4%. Figure 9 displays the comparison between the simulated and measured short-circuit currents of the photovoltaic microgenerator. The simulated results are derived from the short-circuit current data of the photovoltaic microgenerator under different irradiance levels, as depicted in Figure 2. Similarly, the measured results are acquired from the short-circuit current data observed under varying irradiance levels, as shown in Figure 7. As shown in Figure 9, the short-circuit current exhibits a proportional relationship with light irradiance. With increasing irradiance, the short-circuit current demonstrates linear growth. At an irradiance of 1000 W/m^2^, the simulated short-circuit current is 248 µA, while the measured short-circuit current is 233 µA. The comparison of simulated and measured short-circuit currents of the photovoltaic microgenerator reveals an error percentage of approximately 6%. The difference between the simulated and experimental data in Figure 8 and Figure 9 may be attributed to factors including the material doping concentration, manufacturing variations, and environmental influence.

**Figure 10:**
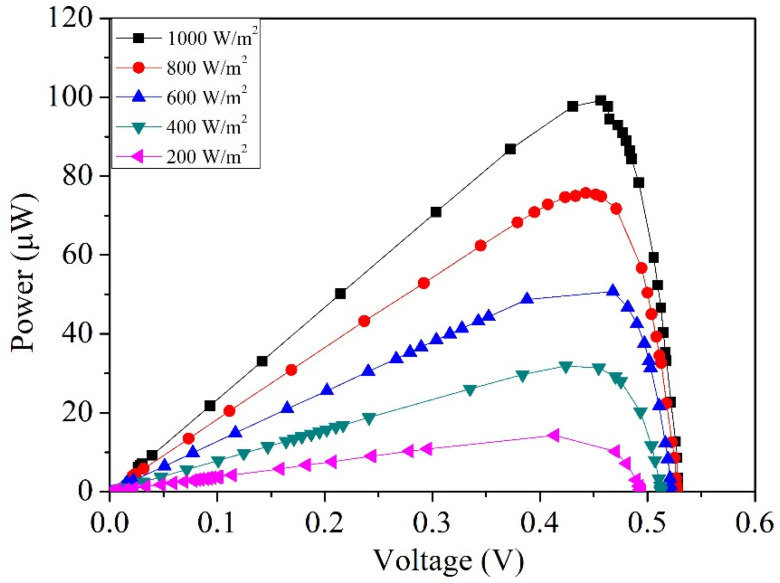
Measured output power of the photovoltaic microgenerator at varying voltages.

**Figure 11:**
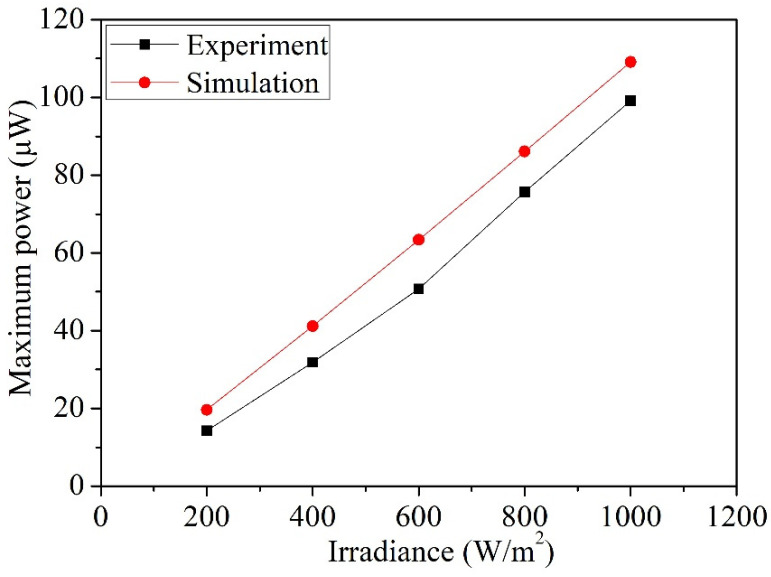
Maximum output power of the photovoltaic microgenerator at different irradiance levels.

By substituting in the measured current and voltage results of the photovoltaic microgenerator from Figure 7 into Equation (1), the calculated measured output power of the photovoltaic microgenerator can be determined. These results are showcased in Figure 10. As shown in Figure 10, at an irradiance level of 200 W/m^2^ and an output voltage of 0.42 V, the photovoltaic microgenerator reaches a peak output power of 11 µW. As the irradiance level escalates to 1000 W/m^2^, the output voltage increases to 0.47 V, leading to a maximum output power of 99 µW for the photovoltaic microgenerator. Figure 11 depicts the comparison between the simulated and measured maximum output power of the photovoltaic microgenerator under different irradiances. The simulated results are based on the maximum output power data obtained from the photovoltaic microgenerator under different irradiance levels, as shown in Figure 3. On the other hand, the measured results are gathered from the maximum output power data recorded under varying irradiance levels, as depicted in Figure 10. The simulated outcomes depicted in Figure 11 show that at an irradiance level of 1000 W/m^2^, the simulated maximum output power of the photovoltaic microgenerator is 109 µW. A comparison between the simulated and measured maximum output power of the photovoltaic microgenerator reveals an error percentage of 10%.

As shown in Figure 7 and Figure 10, at an irradiance of 1000 W/m^2^, the photovoltaic microgenerator has an open-circuit voltage of 0.53 V, a short-circuit current of 233 µA, and a maximum output power of 99 µW. By substituting these values into Equation (2), the measured FF of the photovoltaic microgenerator is 0.8. As depicted in Figure 11, at an irradiance of 1000 W/m^2^, the maximum output power of the photovoltaic microgenerator is 99 µW. The area of the photovoltaic microgenerator is 0.79 mm^2^. By substituting these values into Equation (3), the measured energy-conversion efficiency of the photovoltaic microgenerator is 12.5%.

Table 1 summarizes the performance of various photovoltaic microgenerators. The energy-conversion efficiencies of the photovoltaic microgenerators presented by Sakai et al. [9], Arima et al. [10], Law et al. [12], Hung et al. [16], and Yan et al. [17] are lower than 5%. Horiguchi et al. [13] achieved an energy-conversion efficiency of 7.5%, while Hung et al. [14] achieved 15%. In this study, the energy-conversion efficiency is 12.5%. Compared to those photovoltaic microgenerators [9,10,11,12,13,14,15,16,17,18], the energy-conversion efficiency in this study exceeds that of Sakai et al. [9], Arima et al. [10], Law et al. [12], Horiguchi et al. [13], Hung et al. [15], Hung et al. [16], and Yan et al. [17].

## 5. Conclusions

The photovoltaic microgenerator has been successfully manufactured using the standard 0.18 μm CMOS process, coupled with subsequent post-processing treatment. TCAD was employed to simulate the performance of the photovoltaic microgenerator. The simulated outcomes revealed that, at a temperature of 25 °C and a wavelength of 600 nm, under an incident light irradiance of 1000 W/m^2^, the photovoltaic microgenerator had an open-circuit voltage of 0.55 V, a short-circuit current of 248 µA, and a maximum output power of 109 µW. Following the completion of the CMOS process, a wet-etching post-processing step was implemented to eliminate the surface silicon dioxide layer of the photovoltaic microgenerator. The incident light could directly illuminate the p–n junctions, thereby enhancing the output current of the photovoltaic microgenerator. The experimental results indicated that the photovoltaic microgenerator with the etched silicon dioxide layer exhibited a higher short-circuit current—approximately 25% greater than that of the photovoltaic microgenerator with the unetched silicon dioxide layer. Moreover, measurements demonstrated that at an irradiance of 1000 W/m^2^, the photovoltaic microgenerator with the etched silicon dioxide layer showcased an open-circuit voltage of 0.53 V, a short-circuit current of 233 µA, a maximum output power of 99 µW, a fill factor of 0.8, and an energy-conversion efficiency of 12.5%.

## Figures and Tables

**Table 1:** Summary of performance for various photovoltaic microgenerators.

Authors	Open-Circuit Voltage (V)	Short-Circuit Current	Energy-Conversion Efficiency (%)
Sakai et al. [9]	0.821	5.1 mA	2.35
Arima et al. [10]	0.83	0.4 μA	2.6
Law et al. [12]	0.84	1.25 nA	0.3
Horiguchi et al. [13]	1.3	6 μA	7.5
Hung et al. [14]	0.57	35 mA	15
Hung et al. [15]	2.05	2.6 mA	11
Hung et al. [16]	0.5	11 μA	0.5
Yan et al. [17]	0.53	10.6 mA	4.45
Sichao et al. [18]	0.53	15.3 mA	5.45
This work	0.53	233 μA	12.5

## Data Availability

Data sharing is not applicable.
